# Cyanobacterial ribosomal RNA genes with multiple, endonuclease-encoding group I introns

**DOI:** 10.1186/1471-2148-7-159

**Published:** 2007-09-08

**Authors:** Peik Haugen, Debashish Bhattacharya, Jeffrey D Palmer, Seán Turner, Louise A Lewis, Kathleen M Pryer

**Affiliations:** 1 Department of Biological Sciences and Roy J. Carver Center for Comparative Genomics, University of Iowa, 446 Biology Building, Iowa City, IA 52242, USA; 2 Department of Biology, Indiana University, Bloomington, IN 47405, USA; 3 National Center for Biotechnology Information, National Institutes of Health, 45 Center Drive, MSC 6510, Bethesda, MD 20892, USA; 4 Department of Ecology and Evolutionary Biology, The University of Connecticut, Storrs, CT 06269, USA; 5 Department of Biology, Duke University, Durham, NC 27708, USA; 6 Department of Molecular Biotechnology, Institute of Medical Biology, University of Tromsø, N-9037 Tromsø, Norway

## Abstract

**Background:**

Group I introns are one of the four major classes of introns as defined by their distinct splicing mechanisms. Because they catalyze their own removal from precursor transcripts, group I introns are referred to as autocatalytic introns. Group I introns are common in fungal and protist nuclear ribosomal RNA genes and in organellar genomes. In contrast, they are rare in all other organisms and genomes, including bacteria.

**Results:**

Here we report five group I introns, each containing a LAGLIDADG homing endonuclease gene (HEG), in large subunit (LSU) rRNA genes of cyanobacteria. Three of the introns are located in the LSU gene of *Synechococcus *sp. C9, and the other two are in the LSU gene of *Synechococcus lividus *strain C1. Phylogenetic analyses show that these introns and their HEGs are closely related to introns and HEGs located at homologous insertion sites in organellar and bacterial rDNA genes. We also present a compilation of group I introns with homing endonuclease genes in bacteria.

**Conclusion:**

We have discovered multiple HEG-containing group I introns in a single bacterial gene. To our knowledge, these are the first cases of multiple group I introns in the same *bacterial *gene (multiple group I introns have been reported in at least one phage gene and one prophage gene). The HEGs each contain one copy of the LAGLIDADG motif and presumably function as homodimers. Phylogenetic analysis, in conjunction with their patchy taxonomic distribution, suggests that these intron-HEG elements have been transferred horizontally among organelles and bacteria. However, the mode of transfer and the nature of the biological connections among the intron-containing organisms are unknown.

## Background

Group I introns are distinguished by a conserved secondary structure fold of approximately ten paired elements and the ability to catalyze a two-step splicing reaction in which the intron RNA is removed from the precursor RNA transcript [1]. Because of their ability to self-splice, group I (and group II) introns are referred to as autocatalytic RNAs. The majority of group I introns are found in nuclear rRNA genes and in the plastid and/or mitochondrial genomes of fungi and protists [2]. A smaller number of these intervening sequences are found in phage, viral, and bacterial genomes. In bacteria, group I introns interrupt four different tRNA genes [2], the *recA *and *nrdE *genes of *Bacillus anthracis *[3-6], the tmRNA gene of *Clostridium botulinum *[7], the *thyA *gene of *Bacillus mojavensis *[8], the *RIR *gene of *Nostoc punctiforme *[9], and the large subunit (LSU) rRNA genes of *Coxiella burnetii *[10], *Simkania negevensis *[11], several closely related *Thermotoga *species [12], and the cyanobacterium *Thermosynechoccus elongatus *(strain BP-1, formerly referred to as '*Synechococcus elongatus*') [13]. Group I introns have not yet been found in archaea.

In eukaryotes, group I introns are common in protists except the excavates [14]. These sequences are particularly abundant in fungi, algae, and true slime molds. The widespread, but highly biased distribution of group I introns (i.e., frequent in some taxa such as fungi, but absent from others) suggests they have been transferred horizontally among taxa, and come to reside in different genes. Interestingly, group I introns are sometimes associated with homing endonuclease genes (HEGs) that can invade group I introns to promote efficient spread of the intron/HEG into homologous intron-less alleles [homing, reviewed in [15]]. Briefly, the HEG is expressed and intron/HEG mobility is initiated when the site-specific homing endonuclease (HE) generates a double-stranded DNA break at or near the site of insertion in an intron-less allele, soon after mating between intron-containing and intron-lacking organisms [e.g., [16,17]]. HEGs that are associated with group I introns are categorized into five families by the presence of conserved sequence motifs (LAGLIDADG, His-Cys box, GIY-YIG, HNH and PD-(D/E)XK [18,19]) in the HE proteins.

It is currently believed that most intron/HEG elements follow a recurrent gain and loss life-cycle [20]. In this model, a mobile intron/HEG invades by homing an intron-minus population until it becomes fixed at a single genic site. After fixation, the HEG degenerates and is lost because it no longer confers a biological function. Without the HEG, the intron is lost. Once the population is intron-minus the same intron/HEG element (from another population) may re-invade the same genic site. However, the evolutionary outcome may be different if the HEG or the intron gains a function other than endonuclease or splicing activity, respectively. In a few cases, intron-encoded proteins with dual roles have been reported. For example, in addition to functioning as homing endonucleases, I-*Tev*I, encoded within the td intron of phage T4 acts as a transcriptional autorepressor [21], and I-*Ani*I, a LAGLIDADG HEG encoded within a group I intron interrupting the apocytochrome b gene of *Aspergillus*, function as a maturase [22]. By gaining new biological roles the HEG and/or the intron can avoid becoming redundant and lost [see [23]].

Here we report multiple group I introns in rRNA genes of cyanobacterial strains assigned to the genus *Synechococcus*. A common feature of these introns is the presence of LAGLIDADG homing endonuclease genes in peripheral stem-loop regions of the group I ribozyme. To our knowledge, this is the first discovery of multiple group I introns in a single chromosomal gene of a bacterium (multiple group I introns are also present in at least one phage gene [24] and one prophage gene [25]). We analyze the structure of these newly discovered introns and investigate their phylogenetic history in the context of related introns from bacteria and organelles. In addition, we present a compilation of known group I introns in bacterial or phage genomes that encode HEGs.

## Results and discussion

### Group I introns with LAGLIDADG HEGs in the LSU rDNA genes of *Synechococcus *strains

In an unpublished study on cyanobacterial phylogeny, we sequenced the LSU rRNA gene from 25 diverse cyanobacteria. To our surprise, we found introns in two of the LSU genes, from *Synechococcus lividus *strain C1 and *Synechococcus *sp. C9, both originally isolated from a hot spring habitat in Yellowstone National Park, Wyoming, USA [[26]; see also Table 1]. The LSU rRNA gene of *Synechococcus *sp. C9 contains three group I introns, located at positions L1917, L1931, and L2593 (by convention, the numbering reflects the *Escherichia coli *genic position), whereas the *S. lividus *strain C1 LSU rRNA gene contains similar introns at the L1931 and L2593 positions. All five introns possess a full-length HEG, each containing a single copy of the LAGLIDADG motif. Very few introns have been reported in rRNA genes from other bacterial phyla and this is only the second report of introns in cyanobacterial rRNA genes. The first was for a single group I intron (also with a LAGLIDADG HEG) in the thermophilic cyanobacterium *Thermosynechococcus elongatus *[[13]; Table 1].

**Table 1:** Group I introns in bacteria and phage that encode homing endonuclease genes (HEGs)

HEG family	Organism^a^	Taxonomy^b^	Gene^c^	rDNA insertion site^d^	Intron size (nt)	HE size (aa) ^e^	Functional HEs^f^	Accession number
**LAGLIDADG**								
	* Synechococcus sp. C9	Cyanobacteria	LSU	L1917	743	181		DQ421380
	*Thermotoga subterranea*	Thermotogae	LSU	L1917	774	168		AJ556793
	*Simkania negevensis*	Chlamydiae	LSU	L1931	654	143		U68460
	* Synechococcus lividus (strain C1)	Cyanobacteria	LSU	L1931	675	162		DQ421379
	* Synechococcus sp. C9	Cyanobacteria	LSU	L1931	666	167		DQ421380
	*Thermotoga naphthophila*	Thermotogae	LSU	L1931	699	162		AJ556785
	*Thermotoga neapolitana*	Thermotogae	LSU	L1931	700	162		AJ556784
	*Thermotoga petrophila*	Thermotogae	LSU	L1931	698	162		AJ556786
	Coxiella burnetii	Proteobacteria	LSU	L1951	720	157		AE016828
	* Synechococcus lividus (strain C1)	Cyanobacteria	LSU	L2593	744	189		DQ421379
	* Synechococcus sp. C9	Cyanobacteria	LSU	L2593	748	159		DQ421380
	*Thermosynechococcus elongatus*	Cyanobacteria	LSU	L2593	745	175		AP005376
**GIY-YIG**								
	● *Escherichia coli *phage T4	Phage	sunY/nrdD	-	1033	258	I-*Tev*II	NC_000866
	● *Escherichia coli *phage T4	Phage	td	-	1017	245	I-*Tev*I	NC_000866
	*Bacillus mojavensis*	Firmicutes	thyA		1122	266	I-*Bmo*I	AF321518
	*Bacillus subtilis *phage β22	Phage	thy	-	392	pseudo		L31962
	○ *Bacillus anthracis*	Firmicutes	nrdE (prophage)	-	1102	253	I-*Ban*I	NC_003997
**H-N-H**								
	● T-even phage RB3	Phage	nrdB	-	1090	269	I-*Tev*III	X59078
	*Bacillus *phage SPO1	Phage	DNA pol	-	882	174	I-*Hmu*I	M37686
	*Bacillus *phage SP82	Phage	DNA pol	-	915	185	I-*Hmu*II	U04812
	*Bacillus *phage ϕe	Phage	DNA pol	-	903	181		U04813
	*Escherichia coli *phage ΦI	Phage	DNA pol	-	601	131	I-*Tsl*I	AY769989
	*Escherichia coli *phage W31	Phage	DNA pol	-	601	131	I-*Tsl*I	AY769990
	*Bacillus *phage Spbeta	Phage	bnrdF	-	808	173		NC_001884
	Staphylococcal phage Twort	Phage	nrdE	-	1087	243	I-*Two*I	AF485080
	*Bacillus thuringiensis *phage Bastille	Phage	DNA pol	-	853	188	I-*Bas*I	AY256517
	*Streptococcus thermophilus *phage J1	Phage	Lysin	-	1013	253		AF148566
	*Lactobacillus delbrueckii *subsp. *lactis *phage LL-H	Phage	terL	-	837	168		L37351
**PD-(D/E)XK**								
	*Synechocystis *sp. PCC 6803	Cyanobacteria	tRNA-fMet	-	655	150	I-*Ssp6803*I	U10482

^a ^Organism names. Intron hosts reported in this study are marked with asterisks. Filled circles indicate that homologous introns are found in closely related T-even-like phages [50] and the open circle indicates that homologous introns exist in closely related *Bacillus *species and strains [4], but are not included in this table.

^b ^Classification of organisms follows that of the NCBI (National Center for Biotechnology Information) GenBank.

^c ^The gene in which the intron is inserted.

^d ^The numbering reflects the *Escherichia coli *genic position.

^e ^HE length in amino acids (aa). HE gene fragments are indicated (pseudo).

^f ^Active HE proteins that cut the intron minus target sites.

**Figure 1:**
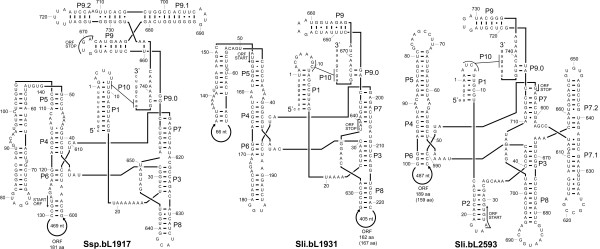
Putative secondary structure of rDNA group I introns in *Synechococcus*. The group I introns are inserted after positions L1917, L1931, and L2593 of the large subunit ribosomal RNA gene. Open reading frames (ORFs) that encode putative homing endonucleases (HEs) with a single copy of the LAGLIDADG motif are inserted into peripheral regions. Paired elements (P1–P10) and every 10th nucleotide position in the introns are indicated on the structures. The L1931 and L2593 introns shown are from *S. lividus *strain C1, whereas the L1917 intron is from *Synechococcus *sp. C9.

The inferred secondary structures of the intronic RNAs are presented for one each of the L1917, L1931, and L2593 *Synechococcus *introns (Fig. 1). Unusual features include open reading frames (ORFs) that extend from peripheral loops into the intron core structure. For example, the L1917 ORF starts in P6 and continues through the group I ribozyme elements P7, P3 and P8 before it stops in P9. The double role of the ORF and ribozyme core regions suggests that these nucleotides must be under strong selective pressure to maintain the catalytic RNA functions and to preserve the genetic code for a functional homing endonuclease. Although uncommon, similar features have been noted in other intron-HEG elements [e.g., [11,27-29]]. It is also noteworthy that the L1917 and L1931 introns are very similar to subgroup IC1 introns that contain a complex P5 region and a classical group IC1 intron P7, but lack a P2 element, which often is associated with long-range tertiary interactions (i.e., with P13 and P14). The L2593 intron has a short P5 region, but contains a relatively large (ca. 65 nt) extension in the P7 region (P7.1 and P7.2) and a short P2. The P7.1 and P7.2 structures were also identified in the crystal structure of a group I intron from the bacteriophage Twort, where it was shown that they are part of peripheral structures that encircle and stabilize the guanosine-binding pocket [30]. Introns lacking the P2 element are common in organelles, and typically belong to the IC2, IA1 or IB4 subclasses of group I introns.

### Compilation of group I introns with HEGs in bacteria and phage

At last count (2005) [see [14,31]], approximately 3% of nuclear group I introns contained a HEG. There are no systematic counts for organellar introns, but in May 2007 the intron database of ref. 2 contained 117 and 83 introns in rRNA and protein genes, respectively, of mitochondria. Of these, 79 contain an HEG, and for 49 introns the presence of ORFs was not determined. In plastids, 105 introns interrupt rDNA genes and 8 interrupt protein genes (note that there are 242 entries of the same trnL intron, and none of these contain an ORF). Of these, 11 contain an ORF and for 80 the presence of an ORF was not determined. Many of the "undetermined" entries do contain ORFs [32], but the exact number remains unclear. In summary, we estimate that at least 50 percent of organellar introns contain ORFs (this value will likely change as more sequence data are added to GenBank).

To assess the frequency of HEGs in bacteria and phage, we searched the literature to determine the total number of published group I introns with HEGs in their genomes. The results of this analysis are summarized in Table 1 and show that the majority of HEGs in bacterial chromosomes belong to the LAGLIDADG family and are found in group I introns located in LSU rRNA genes. Two members of the GIY-YIG family are found, in the chromosomal *thyA *gene (encoding thymidylate synthase) of *Bacillus mojavensis *and in the *nrdE *gene of a prophage of *Bacillus anthracis *and other *Bacillus *species [see Table S2 in ref. [4]]. One catalytically active homing endonuclease (I-*Ssp6803*I), encoded by a group I intron that interrupts the tRNA-fMet gene in the cyanobacterium *Synechocystis *sp. PCC 6803 [28], was recently identified as the first representative of the PD-(D/E)XK family of homing endonucleases [19]. The total number of known group I introns in chromosomal DNA of bacteria (i.e., regardless of whether or not the intron contains an HEG) is currently around 35 if homologous introns in strains of the same species are regarded as one entry (note that about 95 introns are listed at the Comparative RNA web site [2], and that many of these are multiple entries of the same intron in the same species, but in different strains). Therefore, more than 1/3 (14 of 35) of known group I introns in bacteria contain HEGs. Finally, the 14 phage HEGs belong exclusively to the GIY-YIG or HNH families. The three GIY-YIG HEGs are found in *Escherichia coli *phage T4 and in *Bacillus subtilis *phage β22, whereas the eleven HNH HEGs are found in a wide variety of phage. Our study did not involve comprehensive searches of genome databases, but is rather a compilation of known group I introns and HEGs in bacteria. For example, in a recent paper [33] many HNH HEG-like sequences were identified in bacterial and phage genomes, but how many of these are associated with group I introns is unclear. It is likely that more intron/HEG elements remain to be identified in GenBank. His-Cys box HEGs are found exclusively in nuclear introns and are not included in our compilation.

### Phylogenetic analysis of HEG-containing group I introns in bacterial rRNA genes

**Figure 2:**
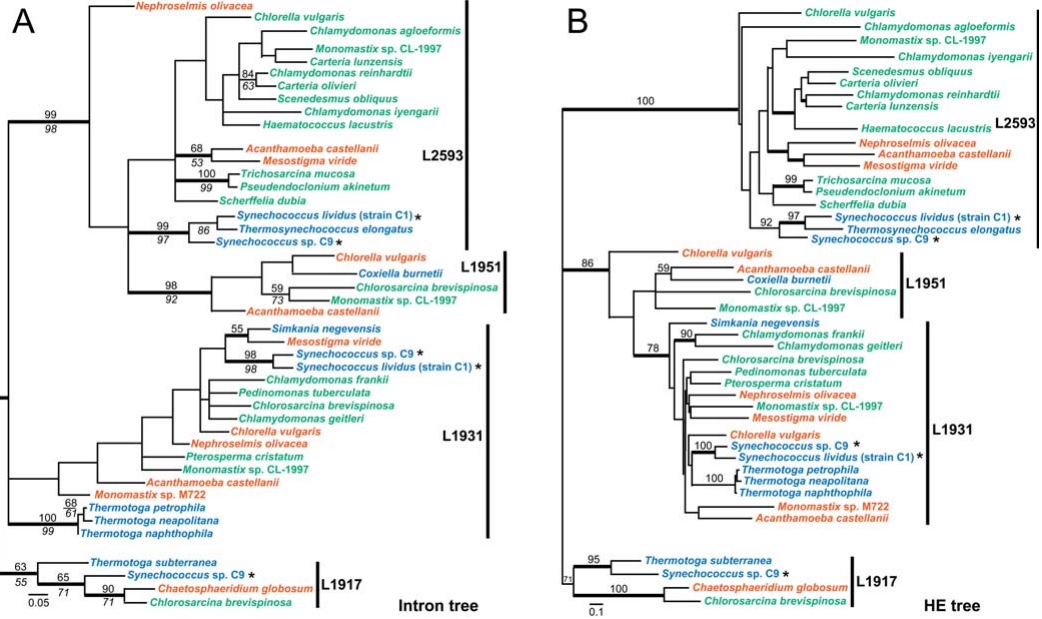
Phylogenetic relationships of rDNA group I introns (A) and their LAGLIDADG HE proteins (B). (A) The 50% majority-rule consensus tree inferred using Bayesian analysis under the GTR + I + Γ substitution model. The tree includes only those LAGLIDADG HEG-containing group I introns that are inserted at the same four rDNA positions (Table 1) at which introns are found in bacteria. The tree is arbitrarily rooted on the branch leading to the L1917 introns. The thick branches denote ≥ 0.95 posterior probability for groups to the right of the values. Numbers above branches indicate minimum evolution (Jukes-Cantor model) bootstrap (BS) values from 2000 replicates, and numbers below branches indicate maximum parsimony values from 200 replicates. Bootstrap support values < 50% are not shown. Vertical bars on the right of the tree mark groups that share insertion positions in the LSU rDNA. Bacterial introns are in blue, chloroplast introns are in green (these are all from green algae), and mitochondrial introns are in vermillion (these are all from green algae, except for the three introns from the amoeba *Acanthamoeba*). Taxa labeled with an asterisk possess the novel introns presented in this paper. The scale bar indicates the inferred number of substitutions per site. (B) Minimum evolution phylogenetic tree of the HE proteins, analyzed under the WAG + Γ substitution model. The tree is arbitrarily rooted on the branch leading to the L1917 HEs. Numbers above the branches indicate the bootstrap support value (from 500 replicates) from a neighbor-joining analysis using the JTT substitution model. Other features of labeling are as in A.

The two unicellular, thermophilic cyanobacterial strains, *Synechococcus lividus *strain C1 and *Synechococcus *sp. C9, are distant relatives based on phylogenetic analyses of small [34] and large subunit rRNA sequences (our unpublished data). We added all five *Synechococcus *intron DNA and HE protein sequences to previously published sequence alignments that contain homologous LSU intron/HEs [32] and inferred phylogenetic relationships among the sequences in these two alignments. HEGs and introns that are inserted at the same rDNA positions are, in general, most closely related to one another [12,32]. Our inferred phylogenetic trees indicate that the *Synechococcus *introns and their HEGs form a cluster with all other known introns or HEs from the same rDNA insertion sites (Fig. 2).

Each of the four, rDNA-positionally-distinct clades of introns/HEGs contains a broad mixture of sequences from bacteria, chloroplasts (entirely from green algae), and mitochondria (mostly from green algae, but with three introns/positions from the amoeba *Acanthamoeba castellanii*) (Fig. 2). These patterns and, crucially, the very restricted and sporadic phylogenetic distribution of these introns (especially so within bacteria and mitochondria, less so within green algal chloroplasts) are consistent with the hypothesis that these introns have been frequently transferred horizontally among and within organelles and bacteria. At the same time, however, because phylogenetic resolution is generally poorly supported within each intron clade (Fig. 2), it is unclear as to how many horizontal transfer events may have been involved in the history of the analyzed introns, much less which clades might have served as donors and/or recipients in any particular horizontal transfer event. Greatly increasing the sampling of these intron families should help address these issues. However, the short length and therefore limited information content of the introns and HEGs will perhaps provide severe constraints on our ability to ever recover a robustly supported phylogenetic history of these mobile genetic elements.

Against this hazy backdrop of likely extensive, but poorly resolved, horizontal transfer it is possible to identify a few lineages of introns/HEs where an element seems to have been transmitted by standard vertical descent once acquired by putative horizontal transfer. Most relevant to this study, the *S. lividus *strain C1 L2593 intron and HE are sister to the *Thermosynechococcus elongatus *L2593 intron/HE, whereas the L2593 intron and HE from *Synechococcus *sp. C9 are sister to this *pair *of sequences. This evolutionary relationship is in agreement with the inferred rDNA phylogeny [see [26] and [34]; our unpublished data], and therefore also with inferred organismal phylogeny. This finding is consistent with the hypothesis that this intron was acquired only once among cyanobacteria and was subsequently subject to strictly vertical transmission. The well-supported sister-group relationship of the L1931 intron and HE from *S. lividus *strain C1 and *Synechococcus *sp. C9 is also in accord with the hypothesis of vertical transmission within cyanobacteria following initial acquisition of the intron via horizontal transfer. In both cases, however, sampling of many additional cyanobacteria, especially those likely to belong to the intron-containing "clades", is needed to better assess the evolutionary history of these introns. Nesbø and Doolittle [12] have likewise concluded that following its putative acquisition from an organellar source, the L1931 intron was subject to strictly vertical descent within a clade of nine intron-containing species and strains of *Thermotoga *(three of which were included in this study; Fig. 2). Finally, the well supported (Fig. 2B) pairing of L1931 HEs from plastid genomes of two chlamydomonads is also consistent with vertical intron descent in this lineage.

### Distribution of single-motif LAGLIDADG HEGs

Group I introns with single-motif LAGLIDADG HEGs are found in biogeographically and phylogenetically distantly related organisms. For example, L1931 introns with single-motif, relatively conserved (Fig. 2B) HEGs are present in 1) *Simkania negevensis *found as a contaminant in a cell culture in Israel [35], 2) the thermophilic bacterium *Thermotoga neapolitana *from submarine hot springs in the Bay of Naples, Italy [36], 3) *Thermotoga naphthophila *from the Kubiki oil reservoir in Japan [37], 4) the cyanobacterium *Synechococcus *spp. from a hot spring habitat in Yellowstone National Park, USA [26], 5) mitochondrial and chloroplast genomes of a diverse array of green algae, and 6) the mitochondrial genome of the amoeba *Acanthamoeba castellanii*. Yet the biological connections (if any) among these organisms and the mode of group I intron transmission remain unclear. *Simkania negevensis *is capable of growing and persisting in acanthamoebal cells [38], indicating a potential association between these two organisms that harbor L1931 introns.

Intron/HEGs are relatively widespread but very sporadically distributed in eukaryotes and prokaryotes. According to the cyclic model for gain and loss of this type of selfish intron [20], the intron/HEG is destined for degradation and loss after a population has been fixed for the intron. However, the intron/HEG can continue to persist by repeatedly spreading into new populations or species via horizontal transfer. The enormous number of prokaryotes on our planet (estimated at 4–6 × 10^30 ^cells [39]) and their presence in virtually every environment compatible with life may provide a constant source of intron-less populations that the intron/HEGs can potentially invade.

Given high rates of horizontal transfer in prokaryotes [e.g., [40,41]], it is surprising that only a small number of introns have been found in their rDNA genes. As of 28 December 2006, 428 prokaryote genomes have been sequenced and another 683 are in progress [42]. In addition, a search of the GenBank nucleotide sequence database [43] limited to nearly complete rRNA gene sequences of known prokaryote origin (i.e., excluding sequences determined from bulk environmental DNA) returned 9,093 records for small subunit rRNA (> 900 nucleotides in length), and 222 records for large subunit rRNA (>2000 nucleotides in length). Even though these numbers overestimate the complete number of prokaryote rRNA gene sequences in GenBank, they provide a rough estimate of how rare rDNA introns are in prokaryotes. It is therefore surprising to find three group I introns with HEGs in a single rDNA gene (in *Synechococcus *sp. C9). It is unclear why *Synechococcus *sp. C9 contains three introns and *S. lividus *strain C1 contains two, whereas the vast majority of bacteria contain no rDNA introns and the few others that have any introns possess only one.

One possible explanation is that the life history and/or physiology of this cyanobacterial group promote intron transfer. Alternatively, introns may sometimes serve a role in the host cell and therefore accumulate in these lineages. Whatever the reason, once inserted into rDNA, introns could pose a risk for bacteria because they could potentially interfere with posttranscriptional processing of precursor rRNA transcripts. Although not fully understood, this processing is relatively complex in bacteria [e.g., [44-46]]. In addition, group I ribozymes catalyze side reactions other than self-splicing, reactions that result in intron RNA circles and fragmented rRNAs [47]. Some rDNA operons and primary transcripts contain many group I introns (e.g., the rDNA operon of the myxomycete *Fuligo septica *harbors 12 group I introns [48]), which makes it increasingly important to strictly regulate group I ribozyme activity towards splicing and not circle formation.

## Conclusion

We found multiple HEG-containing group I introns in cyanobacterial LSU rRNA genes. Specifically, the LSU rRNA gene of *Synechococcus *sp. C9 contains three group I introns, at positions L1917, L1931, and L2593, whereas the *S. lividus *strain C1 LSU rRNA gene contains similar introns at L1931 and L2593. This finding is surprising because the vast majority of bacteria contain no rDNA introns and the few others that have any introns possess only one. The intron-encoded HEGs belong to the LAGLIDADG family, and contain one copy each of the conserved amino acid motif that defines this family (i.e., the LAGLIDADG motif). Phylogenetic analyses show that the cyanobacterial introns and their HEGs are closely related to introns and HEGs located at homologous insertion sites in organellar and bacterial rDNA genes. Finally, from previous studies it is estimated that approximately 3% of nuclear group I introns contain HEGs. In our survey of group I introns and HEGs in the literature we estimate that at least half of organellar group I introns contain HEGs, and that about one third of bacterial group I introns contain HEGs.

## Methods

### Bacterial strains and nomenclature

Axenic slant cultures of *Synechococcus lividus *strain C1 and *Synechococcus *sp. C9 were a gift from David Ward, MontanaState University, Bozeman. These cyanobacterial strains wereoriginally isolated from microbial mat communities in Octopus Spring, Yellowstone National Park, Wyoming, U.S.A. Cells were scraped fromthe slants and DNA was isolated with the Puregene kit (GentraSystems, Minneapolis, MN) following the manufacturer's protocol.

The bacterial nomenclature used in this study is not addressed here other than to point out that the cyanobacterial names in this paper are of botanical origin and have not been validly published under the rules of the Bacteriological Code, unlike the other bacterial names in this report. Therefore they should be considered *ad hoc *and not necessarily consistent with inferred phylogenetic relationships.

### PCR and DNA sequencing

Approximately 2.8 kb of the 23S rRNA gene was amplified from genomic DNA by polymerase chain reaction (PCR) using primers 36F and 2763R [see Additional file 1]. Amplifications were carried out in 50 μL reactions under standard conditions in a PTC 200 DNA Engine thermal cycler (MJ Research). The reaction mixture typically contained 1.0 U of Taq Polymerase and 10× PCR buffer (Gibco BRL Life Technologies), 0.04 mM of each deoxynucleotide, 600 nM of each amplification primer, approximately 50 ng of genomic template DNA, and purified water to volume.

Temperature and cycling conditions were as follows: one 95°C denaturation cycle for 3 min, followed by 35 cycles of 95°C denaturation for 15 sec, primer annealing at 49°C for 15 sec, and elongation at 72°C for 90 sec. Four μL of the amplified products were visualized on 1.5% agarose minigels and the remainder was purified using 30,000 NMWL low-binding, regenerated cellulose membrane filter units (Millipore). Agarose plugs were sometimes taken of weak PCR products and reamplified at 51°C using the same conditions. Both strands of purified PCR products were directly sequenced in 10 μL reactions using the sequencing primers listed in Additional file 1. Cycle sequencing was conducted using dRhodamine Dye Terminator reagents and a PE-ABI 377 automated DNA sequencer (Perkin Elmer – Applied Biosystems). Sequence fragments were edited and assembled into contigs using Sequencher 3.0 (Gene Codes). Sequences obtained in this study have been assigned GenBank accession numbers DQ421379–DQ421380.

### Intron secondary structure prediction and GenBank searches

The central paired elements (P3, P4, P6, and P7) in group I introns were identified by comparing the intron sequences to available secondary structures of related introns (identified by BLAST searches). Secondary structures of peripheral regions were predicted using Mfold [49].

The number of available small and large ribosomal RNA gene sequences of known origin was determined by searching the NCBI (GenBank) databases, restricting the search to prokaryote organisms and excluding sequences determined from bulk environmental DNA. The search was further restricted to complete or nearly complete gene sequences, at least 900 nucleotides in the case of small subunit (16S) rRNA sequences and at least 2000 nucleotides in the case of large subunit (23S) rRNA sequences.

### Phylogenetic analyses

The five *Synechococcus *intron DNA and HE protein sequences were added to previously published sequence alignments [35]. Only intron and HE sequences from homologous LSU positions were kept, and the final alignments contained 44 sequences with 139 nt and 136 aa, respectively [see Additional files 2 and 3]. Phylogenetic analyses were done as previously described [35], and will only be explained here briefly. A minimal evolution tree (WAG + Γ model) was inferred from the protein data set using the programs TREE-PUZZLE 5.0 (to calculate distances), and Fitch (for inferring the topology) from the PHYLIP V3.6a3 program package. TREEVIEW 1.6.6 was used to produce the tree image. Support for nodes was calculated with one bootstrap analysis (neighbor-joining, JTT-model, and 500 replicates), and Bayesian inference (WAG + Γ model, 2 million generations and 50,000 cycles as the burn-in). A 50% majority-rule consensus tree was inferred from the intron data set using Bayesian analysis under the GTR+I+Γ substitution model. The tree includes only those LAGLIDADG HEG-containing group I introns that are inserted at the same four rDNA positions (Table 1) at which introns are found in bacteria. Two sets of bootstrap values were calculated [minimum evolution (Jukes-Cantor model and 2000 replicates) and maximum parsimony (200 replicates)].

## Abbreviations

HEG, homing endonuclease gene; HE, homing endonuclease; LSU, large subunit, rRNA, ribosomal RNA; rDNA, ribosomal DNA; ORF, open reading frame; SSU, small subunit.

## Authors' contributions

PH reconstructed the putative secondary structures of rRNA group I introns in *Synechococcus *strains, carried out the phylogenetic analyses, compiled the list of group I introns with HEGs in bacteria, and drafted the manuscript. DB participated in the analysis and interpretation of the data and in manuscript preparation. JDP conceived of the broader study using LSU rDNA to examine the phylogeny of cyanobacteria, and assisted with data interpretation and manuscript preparation. ST participated in the broader phylogenetic study, in the experimental design that led to the discovery of the introns reported here, in GenBank searches, and in the drafting of the manuscript. LAL assisted with data analysis and interpretation, and in manuscript preparation. KMP planned and coordinated the study, designed primers, sequenced the LSU rRNA data reported here (including the introns and their encoded HEGs), and assisted with drafting the manuscript. All authors have read and approved the final manuscript.

## Supplementary Material

Additional file 1 Oligonucleotides used in this study. Primers used for amplication and sequencing 23S rRNA (including introns and homing endonuclease genes) in *Synechococcus*. Click here for file

Additional file 2 Group I intron dataset. Nexus formatted intron data set. Click here for file

Additional file 3 Homing endonuclease data set. Nexus formatted homing endonuclease data set. Click here for file
