# Supplementary material for: Cyanobacterial ribosomal RNA genes with multiple, endonuclease-encoding group I introns
Source: BMC Evol Biol. 2007 Sep 8;7:159. doi: 10.1186/1471-2148-7-159 (PMC1995217; doi:10.1186/1471-2148-7-159)
Supplement: Additional file 1 — Oligonucleotides used in this study. Primers used for amplication and sequencing 23S rRNA (including introns and homing endonuclease genes) in Synechococcus. [file 1471-2148-7-159-S1.doc]

**Additional file 1.** Primers used for amplification and sequencing of 23S rDNA (including introns and homing endonuclease genes, HEGs) in *Synechococcus*

| Primer name | 5' -> 3' primer sequence |
| --- | --- |
| *23S rRNA*a |  |
| *KP.36F | GCACACAGAGGCGAAGAAGGA |
| KP.479F | AGTACCGCGAGGGAAAG |
| KP.798F | AGGGGTGAAATGCCAATC |
| WL.1186F | GGTAGRRGAGCGTTC |
| WL.1608F | AAACCGACACAGGTRG |
| KP.1910F | GTAACTATAACGGTCCTAAG |
| WL.1930F | GTAGCGAAATTCCTTGTCG |
| WL.2242F | GTTTRACTGGGGCGGT |
| WL.2419F | TCGCTCAACGGATAAAAG |
| KP.248R | CCGTTCGCTCGCCGCTAC |
| KP.591R | TCGCCGGCTCATTCTTCA |
| WL.1091R | AGTGAGCTATTACGC |
| WL.1373R | TACGCYTKTCRGCC |
| VC.1686R | GGGGCCATTTTGCCGAGTTC |
| VC.1929R | CTTAGGACCGTTATAGTTAC |
| WL.2257R | ACCGCCCCAGTYAAAC |
| WL.2419F | TCGCTCAACGGATAAAAG |
| WL.2436R | CTTTTATCCGTTGAGCGA |
| VC.2595R | CGACGTTCTGAACCCAGCTC |
| *VC.2763R | CCCGCTTAGATGCTTTCAGC |
| ***Introns and HEGs in Synechococcus lividus* (strain C1)b** | |
| C1.INT1.F1 | CTAGGAATCCTCAGAGACT |
| C1.INT1.F2 | TGGATTGTTGGTTTTGTTGATG |
| C1.INT2.F1 | CTCGGATGGTGTTGAATGTA |
| C1.INT1.R1 | AACATCAAGTCTCTGAGGA |
| C1.INT1.R2 | AAGCACCCCTCACCATCAACAAA |
| C1.INT2.R1 | GAACATAGGGAGTCAGATTG |
| ***Introns and HEGs in Synechococcus* sp. C9c** | |
| C9.141F | CGGAGGAAAAGAAAACAAC |
| C9.2204F | CCGAGCCGTAGTTGAGAATGTG |
| C9.2268F | AAGCGAGATTTTAGCAAGTTCA |
| C9.2329F | CTGAAGGCGGTTTGCGAGAGAT |
| C9.2365F | AAGGGATGAATGACGGTGGTAA |
| C9.2499F | TGAAGGACTGGTGGGGGTAACT |
| C9.2814F | CGCAGTGGGTCGTTGGTTTTGT |
| C9.2876F | AAGGAAATGGCGGTTGGGTATC |
| C9.2975F | TGTGGGGTGGTGCGGGTCAATC |
| C9.3111F | TTCGGGATGTGGTTTTACTCTG |
| C9.3188F | GCCCAAATGAATCGCAGTCGTA |
| C9.2057R | CGAGCGGGGACGGCAGGAAATA |
| C9.2286R | ACTTGCTAAAATCTCGCTTCTT |
| C9.2390R | GCGTTTACCACCGTCATTCATC |
| C9.2523R | TGAAGTTACCCCCACCAGTCCT |
| C9.2733R | TGTCCCCCTGTCTCGCTCATTT |
| C9.2986R | CACCACCCCACAGCCAAAATAG |
| C9.4598R | AACAGGAGCCGACATTTGAA |
| C9.4661R | ATGGTTCTCCGTATCACT |
| C9.4717R | GGGCTCCTCTCAATACT |
| C9.INT1.F2 | TCCAGACTCAAACCCATACC |
| C9.INT2.F1 | ACATCGGGCGTTTTTAGAGT |
| C9.INT2.F2 | ATTGTGGCGGAACTGAAG |
| C9.INT1.R1 | CAGGCGGTATGGGTTTGAGT |
| C9.INT1.R2 | AGCGGGGACGGCAGGAAATA |
| C9.INT1.R3 | GCCTGCTTCCGTCAAATGCT |
| C9.INT1.R4 | TCATAGGGTCTTTCTGTCCA |
| C9.INT2.R1 | ACGCCCGATGTTTTTGTTTT |

aAll primer numbers approximate *Escherichia coli* LSU rRNA numbering. Asterisk (*) denotes amplification primers. F denotes forward primer; R denotes reverse. KP = primers designed by K. Pryer based on a LSU rRNA alignment of *Synechocystis* PCC 6803 and *Synechococcus* PCC 6301. WL = primers cited in Table 2 of Ludwig et al. (1992). VC = primers cited in Table 1 of Van Camp et al. (1993).

b “INT” primers specifically designed to “walk through” the two *S. lividus* (strain C1) 23S introns and their encoded homing endonuclease genes.

c “INT” primers specifically designed to “walk through” the three *S.* sp. C9 23S introns and their encoded homing endonuclease genes; all other primers designed to improve double-stranded reads based on C9 23S sequence data generated during this project; numbers do not approximate *Escherichia coli* LSU rRNA numbering in this case, but correspond to the nucleotide position in the 4808bp 23S sequence record reported for C9 in GenBank (DQ421380).

**References cited in Additional file 1:**

Ludwig, W., G. Kirchhof, N. Klugbauer, M. Weizenegger, D. Betzl, M. Ehrmann, C. Hertel, S. Jilg, R. Tatzel, H. Zitzelsberger, S. Liebl, M. Hochberger, J. Shah, D. Lane, P.R. Wallnöfer, and K.H. Scheifer. 1992. Complete 23S ribosomal RNA sequences of gram-positive bacteria with a low DNA G+C content. System. Appl. Microbiol. 15:487-501.

Van Camp, G., S. Chapelle, R. DeWachter. 1993. Amplification and sequencing of variable regions in bacterial 23S ribosomal RNA genes with conserved primer sequences. Curr. Microbiol. 27:147-151.
