# Supplementary material for: Cyanobacterial ribosomal RNA genes with multiple, endonuclease-encoding group I introns
Source: BMC Evol Biol. 2007 Sep 8;7:159. doi: 10.1186/1471-2148-7-159 (PMC1995217; doi:10.1186/1471-2148-7-159)
Supplement: Additional file 2 — Group I intron dataset. Nexus formatted intron data set. [file 1471-2148-7-159-S2.doc]

**Additional file 2.** Nexus formatted intron data set. Intron naming follows that of Johansen and Haugen 2001, RNA, 7:935-9361.

begin data;

dimensions ntax=44 nchar=139;

format datatype=dna interleave=yes missing=? gap=-;

matrix

bTsuL1917 AAAGAGTAGTTACGGTACTC GGCTGTATGCGGGAAATCTG GAAGCGCCAGACAACC-CGC AGGAAACTCATCTGATGAGG ATCCTCAGAGACCATAC---

bC91L1917 AGACTGTAGTTACGTAATCT GGCTATATGCTGGAAATCCG GTCAGTGCGGACAATC-AGC AGGAAAGATGCCATAGGCAA ATCCTCAGAGACTATAC---

cCbrL1917 AGACTGTAGTTATAAAATTT AGCTATGTGCTGGAAATCTC AACAGTAGAGACAATC-AGC AGGAAAGACTTTTTACAAGA ATCCTCAGAGACTATAC---

mCglL1917 AAAATGGAGTTGTAAAATTT AGCTATGTGCTGGAAATCTT GTTAGTAAAGACAATC-AGC AGGAAAGACTTTCTGAAAGA ATCCTCAGAGACTATAC---

bTpeL1931 AGCATGCCTGCCCACAGCCC GGCTGTATGCGGGGAACCCT TAGGGATTGGGTAATC-CGC AGGAAACCCCGTGA-GGGGG ATCCTCAGAGACTGTAC---

bTneL1931 AGCGTGCCTGCCCACAGCCC GGCTGTATGCGGGGAACCCT TAGGGATTGGGCAATC-CGC AGGAAACCCCGTTAGGGGGG ATCGTCAGAGACTGTAC---

bTnaL1931 AGCATGCCTGCCCACAGCCC GGCTGTATGCGGGAAACCCT TAGGGATTGGGTAATC-CGC AGGAAACCCCGATA-GGGGG ATCCTCAGAGACTGTAC---

bSneL1931 TAATAGCCTTAGTAAAACCA AACTATATGCGGGAAACCCT CAATATGTGGGCGATC-CGC AGGAAAGACTAATAATTGGA ATCCTCAGAGACCAAAC---

bC92L1931 GAATTGCCTTAGTAAAACTG ACC-ATATGCGGGAATTCCT CGACAGGGGGACAATC-CGC AGGAAAGACCTGGTCCAGGA ATCCCCAGAGACTTAAT---

bC11L1931 TAACGGCCTTAGTAAAACTG ACC-ATATGCGGGAAATCCT CTACATGGGGATAATC-CGC AGGAAAGACCTAGTCTAGGA ATCCTCAGAGACTTGAT---

cCfrL1931 TAAACGCTTTAGTAAAACTG AACTATATGCTGGAAACCCT CAACAGGGGGGCAATC-AGC AGGGAAGGATGTTAAACATA ACCCTCAGAGACTATAC---

PtuL1931c TAAGAGCCTTAGTAAAATCA AACTATATGCTGGAAATCCT CCACATGGGGACAATC-AGC AGGGAAGACTTTCAGAAAGA ACCCTCAGAGACTATAC---

cCbrL1931 TAAAGGCCTTAGTAAAACTA AACTATATGCTGGAAATCCT CCACATG-GGACAATC-AGC AGGGAAGACCAATCATTGGA ACCCTCAGAGACTATAC---

PcrL1931c GAATTGCCTTAGTAAAACTT AACTGTATGCTGGAATTCCT CTACATGGGGACAATC-AGC AGGAAACCGAAGCCCTAGGG ATCCTCAGAGACTAGAC---

MspL1931c GAACGGCCTTAGTAAAACTT AACTATATGCTGGAAATCCT CACTAGGGGGACAATC-AGC AGGAAACCAAAGAGAGTAGG ATCCTCAGAGACTATAC---

cCgeL1931 TAATTGCCTTAGTAAAACTA AACTATATGCTGGAAATCCT CAACAAGGGGACAATC-AGC CGGGAAGGCTTAATTTAAGA ACCCTCAGAGACTATAC---

mAcaL1931 GAATTGCCTTCTCTTTTATT AACCGTATGCCGGAATTCCT CAACACGGGGACAATC-GGC AGGAAACCTTAAAGAAAGGG ATCCTCAGAGACTATAC---

MspL1931m GAATTGCCTG-----TACTT GGCTATTTGCGGGAATTCCC CAACAAGGGGACAATC-CGC AGGAAAGAAAAATTTTTTCG CTCCTCAGAGACTGTAC---

MviL1931m AAAATGCCTTAGTAAAACTA AACTATATGCGGGAAATCTT AAAAAAATAGACAATC-CGC AGGAAAGACTATTAAATAGA ATCCTCAGAGACCAAAC---

mCvuL1931 TAAATGCCTTAGTAAAACTA AACCATATGCTGGAAATTCT CAACACGGGGACAATC-AGC AGGAAAGATTTTTCAAAAAA ATCCTCAGAGACTTTAC---

mNolL1931 TAACAGCCTTATTAAAACCA AACTATATGCTGGAAATCCT CCACATGGGGACAATC-AGC AGGAAACCAAATAAAGTAGG ATCCTCAGAGACTATAC---

cCbrL1951 GAACAGCCCGAATGACGCCA ACTCATAACGGTGAACCCTA AAGTATCATGGGAATA-CCG CGGGAATCTTATGCATTTGA ACCTGTAACGACTGATCTGT

cMspL1951 AAACTGCCCGGTTAATTCCA GCTCATAACGGTGGAATCTA AATCTCCATGATAATA-CCG TGGGAAGATTTATAATAAAT CCCCGTAACGACTGATCTTG

mAcaL1951 AGAATGCCCGATTAACGTCA GCTAATAACGGTGAAACCCT AAAAAATATGGCAATA-CCG TGGGAACGATTATATAATTG ACCCGTAACGACTGAATACA

mCvuL1951 TTTTA????????????TCG ACTCATAACGGGGGAAGCCT AAGAATCATGGTAATC-CCG TGGGAAGTGTGATTATCACA CCCCGTAACGACTGATCGAA

bCbuL1951 AAAAAGCCCCGCTAAACTCA GCTCATATCGGGGGAACCCT CCAGCAAAGGGCAATC-CCG AGGGAAGTCTTAA----TGA CCCCGTAACGACTGATCGTG

cCreL2593 AAATAGCGTGTGCTTGCTCG GCTTATATCGGTGAAACCTT CCAATAGATGGCAACG-CCG AGGGAAGACCATTAAATCGT CCCCGTAGAGACTTTATAGT

cSobL2593 AAATAGCGTTTATCTGTTTG GCTTATATCGGTGAAACCTT CCTGTAGATGGCAACG-CCG AGGGAAGACTCCATTGTCGT CCCCGTAGAGACTTCAGTTT

cMspL2593 AAACAGCGTTTAGTTAGTTG GCTTATATCGGTGAACCCTA AAGCTCCATGGCAACG-CCG AGGGAAGACTTTTGAATCGT CCCCGTAGAGACTGTGAGAT

cCagL2593 TGACTGCGTGTGTTTGCTTG GCTTATATCGGTGAAACCTA CGATTATTTGGCAACG-CCG AGGGAAGACTTTCACAAAGT CCCCGTAGAGACTTCTAGAT

cCluL2593 AAACAGCGTTTGTCTGTTCG GCTTATATCGGTGAACCCTA AAAAGCTATGGCAACG-CCT AGGGAAGACCTCCGTGTCGT CCCCGTAGAGACTTCAGTGT

cColL2593 AAATAGCGTGTGCACGTTCG GCTTATATCGGTGAAACCTT CCAATAGAAGGCAACG-CCG AGGGAAGATCTTTTAAACGT CCCTGTAGAGACTTCAGAGT

cCiyL2593 AAAT-GCGTGTGCACCCTTG GCTTATTTCGGTGAAACCCT ACCATAGCAGGCAATA-CCG AGAGATATTTATAAAAAATT ACTTGTAGAGACTTTATTGT

cTmuL2593 AGAT-GCGTTGGTTGCTTCG GCTTATATCGGTGAAACCTT CTCTAAAAAGGCAATA-CCG AGGGAAGAACCATATTTGTT CCCTGTAGAGACTGAAAGCC

cSduL2593 AGATTGCGTTTATTTTCTTG GCTTATATCGGTGAAACCTT CAAAGAAAAGGTAATA-CCG AGGGAAGAATTTAAAAAATT CCCTGTAGAGACTGAAAATG

bC12L2593 AAATCGCGTCACTTCACCCG ACT-AAATCGGTAGAATCTT AGTAGCTAAGACAATA-CCG AGGGTAAGCGGAGCTCTTGA TCCCGTAGAGACTGAAGGTA

bC93L2593 AAATCGCGTCACTGTGCCCG ACT-AAATCGGTAAAACCTT ATGGGGTAAGGCCATA-CCG AGGGTAAGTAGGAATCCTAA TCCCGTAGAGACTGAGAGTA

cPakL2593 AGAT-GCGTTGGTTAATCCG GCTTATATCGGTGAAACCTT ATCTAAAAAGGCAATA-CCG AGGGAAGAGCAATATTTGTT CCCTGTAGAGACTGAAAACC

cCvuL2593 AAACAGCGTTTATCAATTCG ACTTATATCGGTGAAACCTT CACTAGTATGGCAATA-CCG AGGGAAGATTTTAATAATTT CCCCGTAGAGACTTAGGGAT

cHlaL2593 AAAG-GCGTGTTATTGTTTA ACTGATATCGGTAAAACCTT CCAATAGATGGCAATA-CCG AGGGAAGATTTTAATGTTAT CCCCGTAGAGACTTCAAAGT

mNolL2593 AGAATGCGTGTACACGTCTC GCT-ATATCGGTGAAATCTA ACGAATTATGACAATA-CCG AGGGAAGATTGAATATCAAT CCCCGTAGAGACTGAAGTTC

mAcaL2593 AGATAGCGTTGGTTTGTTTA ACTTATATCGGTGAAGCCCT ACGAAGAGGGGTAATA-CCG AGGGAAAATATAATATTATT ACCTGTAGAGACTGAGGATT

mMviL2593 AAACTGCGTTACTTTATTTA ACTTATATCGGTGAACTCTT TCAGCGAGAGACAATA-CCG AGGGAAAACTAAATTATGTT ACCTGTAGAGACTGAGAAGA

bTelL2593 AAAT-GCGTCACTCCACCCG ACTAAAATCGGTAGAATCTT AATGGTTGAGACAATA-CCG AGGGAAGCAAGGGCTCTTGA TCCCGTAGAGACTGAAGGAC

bTsuL1917 -------GCCGAGCATCGGA TGATGATATGGTCCAATCC

bC91L1917 -------GCCAGACATCTGG TGATGATATAGTCCGGTCT

cCbrL1917 -------GCTAAACGAATTT CGATGATATAGTCCGATCT

mCglL1917 -------GCTAAACTTGACA AGATGATATAGTCCGATCT

bTpeL1931 -------GCCGGGCATCGGA TGAAGATACAGTCCACCCC

bTneL1931 -------GCCGGGCATCGGA TGAAGATACAGTCCACCCC

bTnaL1931 -------GCCGGGCATCGGA TGAAGATACAGTCCACCCC

bSneL1931 -------GTTT-GGAGTAAA CTAAGATAGAGTCCGACTC

bC92L1931 -------GTTCAGGTACCGT ACAAGATAAAGTCCAGCTT

bC11L1931 -------GTTCAGGTGCCGC ACAAGGTAAAGTCCAGCTT

cCfrL1931 -------GTTCAGGAGTGAC TTAAGATAGAGTCCAGCCG

PtuL1931c -------GTTTGGGGATTAT CTAAGATAGAGTCCAGCTC

cCbrL1931 -------GTTTGGAGGCAGC CTAAGATAGAGTCCAGCTT

PcrL1931c -------GTTAAGAAGATTC TTAAGATAGAGTCCGACTG

MspL1931c -------GCTAAGCTCATTG GGAAGATAGAGTCCAACAA

cCgeL1931 -------GTTTGGAAATAAT TTAAGATAGAGTCCAGCTC

mAcaL1931 -------GTTGAATATCGAT -AAAGAAATAGTCCGAACT

MspL1931m -------GCCAAGCATTAAA TGAAGATAAAGTCCGAACT

MviL1931m -------GTTTAGAATTGAA TTAAGATAGAGTCCGACTT

mCvuL1931 -------GTTTGGGGATGAT CTAAGATAAAGTCCAGCTC

mNolL1931 -------GTTTGGGAATGAT TTAAGATAGAGTCCAGCTT

cCbrL1951 CACAACACG-TTGGCACAGT TGAAGGTATAGTCTGATCA

cMspL1951 GCCAAAACG-CTGGCACAGT TGAAGGTATAGTCTGAACA

mAcaL1951 AATAAAACG-CTGACCCGGG TGATGATATAGTCTAAACT

mCvuL1951 GGCAACACG-TCGACCCAGG TGAAGGTATAGTCTGATCT

bCbuL1951 TGCAATACG-CTGACTTAGA AAATGGTATAGTCTACTCC

cCreL2593 ATAATACGC-CGACTCCGGA TGAAGACATAGTCCATGCC

cSobL2593 ATAATACGC-CAACTCTAGA TGAAGACATAGTCCATGCC

cMspL2593 ATAATACGC-CAACTCCGGA TGAAAACATAGTCCATGCC

cCagL2593 ATAATACGC-CAACCCCGGG TGGAGACATAGTCCGTGCT

cCluL2593 ATAATACGC-CGACTCCGGA TGAAGACATAGTCCATGCC

cColL2593 ATAATACGC-CGACTCCGGA TGAAGACATAGTCCATGCC

cCiyL2593 ATAATACGC-CAACCCCGGG TGAAGATATAGTCCATGCT

cTmuL2593 ATAATACGC-CGGCCCTAGG TGAAGATATAGTCCATGCC

cSduL2593 ATCAGACGC-CAACTCCGGA TGGAGATATAGTCCGTGCC

bC12L2593 ATCAAATGT-CGGCTCCGGA TGAAGATATAGTCCATGCT

bC93L2593 TTCAAATGT-CGGCTCCGGA TGAAGATATAGTCCATGCC

cPakL2593 ATAATACGC-CGGCCCTAGG TGAAGATATAGTCCATGCC

cCvuL2593 ATAACACGT-CGACTCCGGA TGAAGATATAGTCCATGCC

cHlaL2593 ATAAAACGT-TAACTCCGGA TGAAGATATAGTCCATGCC

mNolL2593 TTCATACGC-GAGCTCCGGA TGAAGATATAGTCCATGCA

mAcaL2593 ATAATACGT-TAACTCCGGA TGAAGATATAGTCCATGCC

mMviL2593 ATAATACGT-TAACCCCGGG TGAAGATATAGTCCATGCT

bTelL2593 ATCAAATGT-CGGCTCCGGA TGAAGATATAGTCCATGCT

;

end;
