# Supplementary material for: Cyanobacterial ribosomal RNA genes with multiple, endonuclease-encoding group I introns
Source: BMC Evol Biol. 2007 Sep 8;7:159. doi: 10.1186/1471-2148-7-159 (PMC1995217; doi:10.1186/1471-2148-7-159)
Supplement: Additional file 3 — Homing endonuclease data set. Nexus formatted homing endonuclease data set. [file 1471-2148-7-159-S3.doc]

**Additional file 3.** Nexus formatted homing endonuclease data set. Intron naming follows that of Johansen and Haugen 2001, RNA, 7:935-9361.

begin data;

dimensions ntax=44 nchar=136;

format datatype=protein interleave missing=-;

matrix

CbrL1951c LYMVLDPWWI-TGFSDGEGC FHISFEKNPI-R-KLQFRVS FSISQNASSVSAIQKIAICF GTGKTQNIRDRHTVKYETRN VDHIVNSIIPHFEKYPLQSN

MspL1951c KNTFDFASWLV-GFIDGEGC FCLSFSERERLTLKIEVRPS FSVSQNSKSMKILETMNTYF GCGGVRHSKEN-THKYEVRN LNHLTTVIIPFFRKYPLLTN

AcaL1951m EKALIDPFWI-SGFVDGEGC FCISFNLKERLTLGIEVRPS FSISQ-TRDLRCLQDFLNFF DCGFIRFSKDN-TWKYECRD LSDIRSKVLPHFEKFTLRTK

CvuL1951m -MKKITSDYIV-GFVDGEGC FTLHIIKKKQSAFGFYFTPS FSVSQNTNSVRVLQDIQQFF HCGFIRNDK-K-TSKYEVRD LKNLTTYIIPFFKKNKLRTM

CbuL1951b MYLHLEP-WYVSGLTEGEGC FSISFNFRKKLKIGIETRPS FSITLNQRDLPLLKEIHSFF ECGAIRFSRDR-TYKYEVRS VKDLVKRIIPHFDNYPLRGG

TpeL1931b --MDLKPDWVV-GFVDGEGC FYVGVNRNRTMKTGYQVLPE FRIVQHERDIQVLYALRKFF GCGVVRRNHDR--YELRIRK RSCLKKVVE-FFEKHPLKTK

TneL1931b --MDLKPDWVV-GFVDGEGC FYVGVSRNRTMKTGYQVLPE FRIVQHKRDIQVLYALRKFF GCGVVRKNHDR--YELRIRK RSCLKKVVE-FFEKHPLKTK

TnaL1931b --MDLKPDWVV-GFVDGEGC FYVGVNRNRTMKTGYQVLPE FRIVQHERDIQILYALKKFF GCGVVRKNHDR--YELRIRK KSCLKKVVE-FFEKHPLKTK

SneL1931b --MHLDAQWIV-GFVDGEGC FKVSLNK---MK---EIETE FTVVQNEQDVQVLFALKKHF GCGVVRKIPNR--MCYRVKE TKHLLTKIIPFFEKHSLKTF

C92L1931b RLMNLETQWVV-GFVDGEGC FHVSINPHKEMAVGYQVLPE FTVVQHQRDVQVLHGLKAYF GCGVVRVNHDR--MAYRVRS IEHLAQRIVPFFLKHPLKTS

C11L1931b RQMDLDVQWIV-GFVDGEGC FHVRVNPHRQMAIGYQVLPE FTVVQHRRDVKVLYALKAYF DCGVVRVNHDR--MAYRVRS IHHLAERICPFFIKHSLKTS

MspL1931c --MSMHEQWI-TGFTDGEGC FHIGISKNNETKLGSQVLLE FVITQHQRDEQLLNEIKNYF GVGVVRKSNDI--LCYRVRS QKHLRDVILPFFEKNILRTK

CgeL1931c MTTKLNAQWIV-GFTDGEGC FNLDVHIKNDMRWGLQMQPE FTVVQNSIDIQILHALKDYF GCGTVSVNRDK--TGTRYKS VKDLHEKIIPFFEQHQLKTK

CfrL1931c --MNIDPQWIV-GFVDGEGC FYVGVLKSNKLKYGYQIQPE FTVVQHELDINVLHALKDYF QVGVVQVNHDR--YAYRVKN LDHFLNVIIPFFETHKLKTK

PtuL1931c --MNLEAQWIV-GFVDGEGC FHIAINSNQEMKLGVQVLAE FVVVQHESDRQILEALKAFW GCGVVRKNNDC--LCYRVRD LEQLKTRIVPFFEKHKLKTR

PcrL1931c --MNLDAQWVV-GFVDGEGC FHIGINNNASMKLGVQVLPE FTVVQHEIDEQVLYALKAHF KCGVVRKNHTR--LAYRARG QENLLKTVIPFFEKHQLKTR

CbrL1931c --MNLEPQWIV-GFVDGEGC FFVGFNKQSTMKIKIQVLPE FTVVQHQRDIAVLQNLKTYF QCGVVQRNHDR--YAYRVRG HENLLKKIIPFFEKHKLKTK

MviL1931m --MKLETQWIV-GFVEGTGC FHVSINPNLEAR--QSLLPE FSVVQHKKNVQILYALKTYF CCGVVQGD-NV--MIYRVRN LNHLTKIIIPFFEKHLLKTK

MspL1931m --MKLTPDWIC-GFVEGEGT FAISLEKNENMKMKMQVRLI FKITQHIKNVQVLYAIKKYF GIGQVKPQNDI--WEYRVSN FEQITNTVIPFFEKHSLHTS

CvuL1931m --MNLQTQWIV-GFVDGEGC FHVAINSQKTSKLAFQVLPE FTITQHKRDVQVLYGLKNFF GCGVVRVNHDR--YCYRVRG FPHLRDIILPFFEKHKLLTK

NolL1931m --MNLNAQWIV-GFVDGEGC FHIEMNPQPSMKMKCQILCS FVITQHIRDIQLLHAIKDYF GCGVVRRDKNI--YCYRVRS FQHLRTIIIPFFEKHELKTK

AcaL1931m --MKIDKNWIV-GFVDGEGC FYIGINKSVDSKLGYQVLPE FRVVQHKRDIKVLYAIKDFF GHGVVCNKSSEI-YEYRVRK FETLHDVILPFFESNGLLTS

CbrL1917c QVPPDKGYYIA-GFVDGEGS FYISARKRKDYSSGW--KFT VHFNVSNADIAVLQVCKKYL GSGEIREPRPGF-YVLEVTD KQKLKTFIVPFFKKFGLSNK

C91L1917b PIPPEIGYYLA-GFTDGEGS FNISCRPRSDYLIPW--KIS VSFNVSQKDRVILALFKRYL GCGTLRGRPDGV-WYYEVTQ LRAVVENVIPFFERFPLSAK

TsuL1917b ---------MA-GFADGEGS FMVVVRKKDDYKSGW--KIS VAFNVSNKDKVVLALFKRYL NCGTLRQRKDGV-WYYEVGN FNAIVENVIPFFDRFRLSAA

CglL1917m QVPPDKGHYIA-GFVDGEGS FYISARKRTDYLSGW--SFE LHFNISNRDLAIMQICKKFL GCGLIRQTRPGF-YTLEVEN RKTLSTYIIPFFKKFGLSNK

ICreIL259 KYNKEFLLYLA-GFVDGDGS IIAQIKPNQSYKFKHQLSLA FQVTQKTQRRWFLDKLVDEI GVGYVR-DRVSDYILSEIKP LHNFLTQLQPFLK------L

SobL2593c IIDKDELIYLA-GFIDGDGS LIAQMVRRHDYKFKYQIKCT VQITQLKKRRHFLEKIQESI GYGIIR-DRISDYVLVEPKC VYWLLKQLSPFLR------L

MspL2593c TLQPTEAAYIA-GFLDGDGS IYAKLIPRPDYKIKYQVSLA ISFIQRKDKFPYLQDIYDQL GRGNLRKDRIADYTIIGSTH LSIILPDLVPYLR------I

CagL2593c NLNETELAYIA-GFLDGDGT IQISTASTERYPFKFQLNVA F--IQLAKRQSFLIDLQQKF GRGNIRK-KVSELVIGDITI IKELLELLLPYLR------T

CluL2593c KFTPDQLLYLA-GLIDGDGS IIAQLVSRKDYTWEFQIRLT VQVTQLKKRRWFLEELQKEI GAGSVR-DRVSDYILTETSN VYKFLKDLQPHLR------L

ColL2593c DLQEKDLIYLA-GFIDADGS IFAQLISNNDYKFNYQIRVT VQITQLTKRKLFLTHIRDLI GVGTIR-DRVSDYVLVEPRF VYKLLTQLQPFLR------L

CiyL2593c TLSPTEAAYLA-GVIDSDGA IIAQIKQNVGYVMLHQL--S VT-LQVTQKVIFLQHIVDLI GEGNLR-DRSKKVVPVSDES IAAVLTQIKPYLV------V

TmuL2593c KLTPEELCYLA-GFLDGDGC INAQIVRRSDYKLKFQIRVS ITFFQKTNRHWFLIWLDKKL DCGTLRK-RMSEYAIIGIAS VRNILLLFKPYLK------L

SduL2593c KLQENELAYIA-GFIDGDGC INAQIIRRVDYKLKFQIRFS VTIIQKTSRHWVVLWFQKKL GCGQIRK-RISEYSLVGKND VQNFLQLIKPFLK------V

PakL2593c KIIPEELSYLA-GFLDGDGC INAQIVRRSDYKLKFQIRVS ITFFQKTNRHWFLIWLDKKL DCGTLRK-RMSAYAITGIAS VRNLLSILKPYLK------L

ICvuIL259 FHDQLKFAWLA-GFVDADGC INAQIVSREDYLLKYQVRVS LTVFQSTTQHFILLDIQKIL GCGTVRK-RMSEFCVVGGTS LQTTLEKLLPYLQ------L

HlaL2593c HLTNEQKAYLA-GFIDCDGS LMAQIVRKPDYAYKFQIRVT IQLSQRTSRIHFLKEIASEV GYGYVV-SRMSDYVITQANI VYELLSLLLPYLR------M

NolL2593m QVTSEQKSYI-SGFLDGDGC ILAQIVRQSCSPYGFQIRVG IHFYQKTSRSWHLEGLKKML GYGTIRK-RISEYSIVGFSA VKLILEALLPHII------M

AcaL2593m KLNNEQLAYLA-GFVEADGC FLVQIIPGLQYRYKHTIRIS IVFYQKKDKHWYFLQLKNLI GLGSIRF-RMLEYSITGLSL VNKFLEMLFPYLI------L

MviL2593m LIDEKVCAYIA-GFLDGDGC LMAQIIKGV-YKYKFRIRLS IVFYQHAKRKWFLLQLKKLF DIGYVRIHKMCDYTITGSRS IEHILLQLMPYIQ------L

TelL2593b VTDNLTLSYIA-GFLDADGC INLQLVRRKDYVLGYQIRAS ITFFQPSSHRSFLEWLKSIF QVGFVR-DRVSEYAIVDTPS VLDVLVTLRSYLK------I

C93L2593b MSSPEVLSYIA-GFLDGDGS INVQLVRRKDYRLGYQIRPS ITFFQKQKHRAFLEWLQSVF SVGCIR-ERMSEYSVVDVKS VERILTQLQPYIR------L

C12L2593b VTDVQTYSYIA-GFLDGDGC INLQLVRRKDYVLGYQIRAS VTFFQRTVHRSFLEWIQSVL GAGFIR-ERVAEYSITESRM VLNVLSNLTPYVR------L

CbrL1951c KQYDFEKFKEICFFIKSEKP LSKESKLLILYLSFEM

MspL1951c KQKDFLLFDQACMIIKENRH LSKEGLKEILQLAAQM

AcaL1951m KLRDFELFKDVVNSVASKQH LNEVGLKRIIDISYQI

CvuL1951m KNKDFQIFCEICGLLNQKQH YTVSGAQKLLDLAFSM

CbuL1951b KQKDFRYFSEICKKIHTNFH LNKQHLIEIIKMAYLM

TpeL1931b KNVDFKKFRRIILMMERGEH LTKEGLIKILEIAMEI

TneL1931b KNVDFKKFRRILIMMERGEH LTKEGLIKILEIAMEM

TnaL1931b KNVDFKKFRRIILMMERGEH LTKEGLIKILEIAMEM

SneL1931b KRVEFQKFRKILLKIQRNEH MSADGIDEIQNIIRQV

C92L1931b KNIDFLKFRDVVLLCQAGEH LTEAGIIKIQQITAQM

C11L1931b KSIDFLKFRDVVLLCQSGDH LKEEGLLKIQRIASQM

MspL1931c KKFAFQRFKQALVLIENKEH LTLEGLDKLRKLRDQV

CgeL1931c KQIEFKRFRNIVRLMNEGYH RVSLKNFLEIVDKGVE

CfrL1931c RRVEFLRFRKICLLLKAGRH LESEEGFSEVLLAKNL

PtuL1931c KRIDFDTFRDVIALMDQQAH LTLEGVEKINQ-IRKT

PcrL1931c KRVDFQKFRKVLLMMEKGEH LTKDGLEKFVK--SSN

CbrL1931c KRVDFEKFRDIVLLMEKKAD LQFEGLEKITKIANTM

MviL1931m QRIAFQKFRTILLKMERKEY LTVEGFEKIKTLSKDL

MspL1931m KKYDFLRVRYVSILIKRGDH LKEDGFLKIVKLRSRM

CvuL1931m KRVDFEKFRKVILKMNCGDH LTPQGIDEIRKIQETM

NolL1931m KHVDFLKFRKVILMMEKKLH LEKEGFERIQQIVLAG

AcaL1931m KKFNFLAFRDVILIMKRREH LTESGLSKIIDIKSRM

CbrL1917c KKAEFRIFRQALRALEGVIR SETELHDFLVLRQKLN

C91L1917b KKRDFSKFKQIVALMQRQAH LTEGGLREILSIREGM

TsuL1917b KRNSYSKFKKVVEIIGRSEH TTKEGIEKILSLRNQM

CglL1917m KKTEFRIFQHALILLEQGIN TPKNLEQLLQLRKRLN

ICreIL259 KQKQANLVLKIIWRLPSAKE SPDKFLEVCTWVDQIA

SobL2593c KKKQADLIIRIIEQLTSSKN SAVLFVQLCRLTDQVA

MspL2593c KKKQANRILHIINLYPQAQK NPSKFLDLVKIVDDVQ

CagL2593c KKRQAAIALLIIDHIQDPV- NCNNLTCVEYL--RRA

CluL2593c KQKQANLVLRIIEQLPSSKA SKEIFLELCNVVDHVA

ColL2593c KKKQANLVLKIIEQLPSSKD SQPEFLKLCKQVDQIA

CiyL2593c KKEQAVLVEQIITALKDNQK DPALFIPICRLVDRVA

TmuL2593c KKRQAILLLKVIEKMPSTQN DPQAFFKLCEQVDQFS

SduL2593c KRRQAQLILEICQQI-SKEQ DPQSFVKLCERVDSIA

PakL2593c KKRQAILLLKIIEKMPHIQN DPQLFLKLCEQVDHFS

ICvuIL259 KRAQAKLVLQIIKKLPNTK- DPSVLMEAALLADKVG

HlaL2593c KVKQANLILKIIQELPSAKV SKDKFIELCILANQVS

NolL2593m KRKLASLVLQIIELHSKVQ- TADDFLQVCYLVDKSA

AcaL2593m KKNLAVLIFRIIKGLNDVKN -EAGFLEVCKLVDEVA

MviL2593m KKTSAALMLQLIKKEKLVT- TKADFIEVCQLVDKIA

TelL2593b KKPQCDLAITVLQEVVHSRL TPEQFLKLAQKVDRFG

C93L2593b KQKQCDLMLEIVAELKHSAL SPQDFLALAKKVDVFE

C12L2593b KKMQCNLVMSVLEEMLSSKL EPAHFLRLARQVDRFG

;

end;
